# Interactions of histatin-3 and histatin-5 with actin

**DOI:** 10.1186/s12858-017-0078-0

**Published:** 2017-03-06

**Authors:** Edna Blotnick, Asaf Sol, Gilad Bachrach, Andras Muhlrad

**Affiliations:** 1 Institute of Dental Sciences, Hebrew University-Hadassah School of Dental Medicine, Jerusalem, Israel; 2 Department of Medical Neurobiology, Institute for Medical Research-Israel–Canada, Hebrew University of Jerusalem, Jerusalem, Israel

**Keywords:** Histatin-3, Histatin-5, G-actin, F-actin, Actin polymerization and bundling, Transglutaminase cross-linking

## Abstract

**Background:**

Histatins are histidine rich polypeptides produced in the parotid and submandibular gland and secreted into the saliva. Histatin-3 and −5 are the most important polycationic histatins. They possess antimicrobial activity against fungi such as Candida albicans. Histatin-5 has a higher antifungal activity than histatin-3 while histatin-3 is mostly involved in wound healing in the oral cavity. We found that these histatins, like other polycationic peptides and proteins, such as LL-37, lysozyme and histones, interact with extracellular actin.

**Results:**

Histatin-3 and −5 polymerize globular actin (G-actin) to filamentous actin (F-actin) and bundle F-actin filaments. Both actin polymerization and bundling by histatins is pH sensitive due to the high histidine content of histatins. In spite of the equal number of net positive charges and histidine residues in histatin-3 and −5, less histatin-3 is needed than histatin-5 for polymerization and bundling of actin. The efficiency of actin polymerization and bundling by histatins greatly increases with decreasing pH. Histatin-3 and −5 induced actin bundles are dissociated by 100 and 50 mM NaCl, respectively. The relatively low NaCl concentration required to dissociate histatin-induced bundles implies that the actin-histatin filaments bind to each other mainly by electrostatic forces. The binding of histatin-3 to F-actin is stronger than that of histatin-5 showing that hydrophobic forces have also some role in histatin-3- actin interaction. Histatins affect the fluorescence of probes attached to the D-loop of G-actin indicating histatin induced changes in actin structure. Transglutaminase cross-links histatins to actin. Competition and limited proteolysis experiments indicate that the main histatin cross-linking site on actin is glutamine-49 on the D-loop of actin.

**Conclusions:**

Both histatin-3 and −5 interacts with actin, however, histatin 3 binds stronger to actin and affects actin structure at lower concentration than histatin-5 due to the extra 8 amino acid sequence at the C-terminus of histatin-3. Extracellular actin might regulate histatin activity in the oral cavity, which should be the subject of further investigation.

**Electronic supplementary material:**

The online version of this article (doi:10.1186/s12858-017-0078-0) contains supplementary material, which is available to authorized users.

## Background

Histatins are histidine rich cationic peptides synthetized in the parotid and submandibular salivary glands and released into the saliva. Histatin-1, histatin-3 and histatin-5 are the main histatin species comprising more than 80% of histatins in the saliva. All histatins are the products of two genes, one is responsible for the production of histatin-1, the other for the production of histatin-3. The other histatins are proteolytic derivatives of the above two histatins. In this work we studied the interactions of histatin-3 and −5 with actin. Histatin-5 is a derivative of histatin-3 it is identical with the N-terminal 24 amino acid sequence of histatin-3, which contains an additional 8 amino acid C-terminal sequence [1]. Histatin-3 and −5 are polycationic peptides, each have five net positive charges derived from lysine and arginine residues. They also contain 7 histidine residues with pK 6.5. The histidines are positively charged at slightly acidic pH, their charge decreases with increasing pH. Histatins are strong antifungal agents. Histatin-5 has the highest activity against pathogenic fungi, such as *Candida albicans* and other medically important *Candida* species [1–3]. Histatin-3 and −5 contain an Amino Terminal Copper and Nickel (ATCUN) tripeptide sequence at their N-terminus. The sequence of the motif in histatins is Asp-Ser-His. The copper complex of the motif generates reactive oxygen species, which has an important role in the antifungal activity of histatins [4]. Histatins also have a positive role in wound healing in the oral cavity [5].

Actin is a negatively charged structural protein and the most abundant protein in the eukaryotic cells. Actin, released from dead cells, is also present in the saliva [6]. Actin has important roles in cytoskeleton formation, cell division, motility, adhesion, signaling and more [7]. Actin exists in either monomer globular (G) or polymer filament (F) form, which are interconvertible into each other. Actin interacts with positively charged proteins and peptides including lysozyme [8], LL-37 [9], eukaryotic elongation factor 1 alpha (eEF1A) [10] and histones [10–12], which polymerize G-actin to F actin and bundle F-actin into actin bundles. F-actin forms bundles with polycationic peptides and proteins mainly via non-specific electrostatic interactions by eliminating repulsion between negatively charged filaments [13]. Specific actin binding proteins bundle F-actin by cross-linking the filaments through attachment of their two discrete actin-binding sites to actin protomers located on separate filaments [14]. Actin bundles are involved in the formation of the high viscosity sputum in cystic fibrosis [15], which is a primary cause of pathology in this disease.

Here we studied the interactions of actin with histatin-3 and −5 and found that they polymerize and bundle actin mainly by electrostatic interactions and affect actin structure. The binding of histatins on actin was studied by limited proteolysis and cross-linking. The main binding site for histatins on actin is Gln 49 located on the D-loop. The interaction of histatins with actin may have an effect on the fungicide and wound healing activity of histatins, and might hamper these health promoting actions during infection induced cell necrosis in which extracellular actin is released. These effects should be subject of further investigations.

## Methods

### Materials

Deoxyribonuclease 1 (DNase1), ATP, ADP, dithiotreitol (DTT) and cysteamine were purchased from Sigma Chemicals Co. (St Louis, MO). Pyrene maleimide, dansyl ethylenediamine (DED) and tetramethyl rhodamine cadaverine (TRC) were obtained from Molecular Probes (Eugene, OR). Histatin-3 (DSHAKRHHGYKRKFHEKHHSHRGYRSNYLYDN), tetramethylrhodamine labeled histatin-3 (Fl-histatin-3), histatin-5 (DSHAKRHHGYKRKFHEKHHSHRGY), FAM labeled histatin-5 (Fl-histatin-5), LL-37 (LLGDFFRKSKEKIGKEFKRIVQRIKDFLRNLVPRTES) and tetramethylrhodamine labeled LL-37 (Fl-LL-37) peptides were purchased from Genemed Synthesis Inc., (San Antonio, TX). The peptides were purified by HPLC (greater than 90% purity) and were determined by Mass Spectrometry. Acetone dry powder prepared from the back and leg muscles of rabbits was purchased from Pel-Freez Biologicals (Rogers, AR). Yeast cofilin and bacterial transglutaminase (TGase) were generous gifts of Prof. Emil Reisler, Univ. of California Los Angeles CA. and Prof. György Hegyi, Eötvös Loránd University, Budapest, Hungary, respectively.

### Preparation of actin

CaATP-G-actin was prepared from acetone dried powder derived from the back and leg muscles of rabbits by the method of Spudich and Watt [16]. CaATP-G-actin was stored in a buffer containing 5 mM TrisHCl, 0.2 mM CaCl_2_, 0.2 mM ATP, 0.5 mM β-mercaptoethanol, pH 8.0 (CaATP-G-buffer). MgF-actin was polymerized from CaATP-G-actin by 30 min incubation with 2 mM MgCl_2_ at 22 °C. MgF-actin was diluted for further treatments in MgF-buffer containing 5 mM 3-(N-morpholino) propanesulfonic acid (MOPS), 2 mM MgCl_2_, 0.2 mM ATP and 0.5 mM DTT, pH 7.4. The concentration of G- and F-actin was determined using the extinction coefficient E^1%^_290_ = 11.5 cm-^1^. (The optical density of actin was measured in the presence of 0.5 M NaOH, which shifts the maximum of absorbance from 280 to 290 nm). Molecular masses: skeletal actin (42 kDa), yeast cofilin (15.9 kDa), DNase1 (31.3 kDa), histatin-5 (3.04 kDa), Fl-histatin-5 (3.39 kDa), histatin-3 (4.1 kDa), Fl-histatin-3 (4.47 kDa), LL-37 (4.5 kDa) and Fl-LL-37 (4.87 kDa).

### Pyrene labeling

Labeling of Mg-F-actin at Cys-374 with pyrene maleimide was carried out according to Kouyama and Mihashi [17] with some modifications. CaATP-G-actin was filtered through a PD-10 column (GE Healthcare) equilibrated with β-mercaptoethanol-free CaATP-G-buffer. After filtration, G-actin (1 mg/ml) was polymerized by 2 mM MgCl_2_ and 100 mM KCl at 22 °C for 30 min, and reacted with pyrene maleimide (16 μg/ml) on ice, for 1 h. The reaction was terminated with 1 mM DTT. Labeled F-actin was centrifuged at 129,151 x g for 2 h; the pellet was resuspended in Ca-ATP-G-buffer and depolymerized by dialyzing in this buffer for 36 h at 4 °C with twice changing the buffer. Finally, actin was centrifuged again at 129,151 x g for 2 h. The supernatant contained the purified pyrene-labeled CaATP-G-actin. The concentration of modified actin was determined by the procedure of Bradford [18] using unmodified actin as a standard. The extent of labeling was determined by using pyrene extinction coefficient E_344_ nm = 22,000 cm^−1^M^−1^, was ~100%.

### Tetramethyl rhodamine cadaverine (TRC) labeling

Actin labeled with TRC at Gln41 (TRC–actin) was prepared by incubating 100 μM CaATP-G-actin with 150 μM TRC and 0.18 mg/ml of bacterial transglutaminase in CaATP-G-buffer, on ice, for 48 h. Labeled G-actin was filtered through a PD-10 column to remove excess of TRC reagent. The concentration of labeled actin was determined by Bradford’s method [18]. The extent of labeling was determined by TRC extinction coefficient E_514_ nm = 78,000 cm^−1^M^−1^, was ~32%.

### Dansyl ethylenediamine (DED) labeling

Actin labeled at Gln-41 with DED was made by adding 100 μM DED and 0.18 mg/ml bacterial transglutaminase to 60 μM CaATP-G-actin. This mixture was incubated in CaATP-G-buffer supplemented with additional 0.2 mM ATP, on ice, for 2 days. Excess of DED reagent from labeled G-actin was removed by gel filtration on a PD-10 column. The concentration of modified actin was determined according to Bradford [18]. The extent of labeling was measured by using DED extinction coefficient E_334_ nm = 4800 cm^−1^M^−1^, was ~40%.

### Fluorescence and light scattering measurements

Actin polymerization was followed by fluorescence increase (at 365 nm excitation and 386 nm emission wavelengths) of pyrene-labeled G-actin [17], which was added to unlabeled G-actin in 10–15%. The kinetics of bundling of MgF−actin was monitored by increase in light scattering, with both excitation and emission wavelengths adjusted to 450 nm [18]. All fluorescence and light scattering measurements were carried out at 22 °C.

### Monitoring polymerization or bundling by sedimentation

Extent of actin polymerization and bundling was measured by high speed centrifugation at 129,151 x g for 2 h and by low speed centrifugation at 20,800 x g for 8 min, respectively. The centrifugations were carried out at 4 °C. The supernatants were separated by 12% sodium dodecyl sulfate polyacrylamide electrophoresis (SDS−PAGE) and analyzed by densitometry using the TINA 2.07d software.

### Transglutaminase (TGase) crosslinking

Actin, histatin-3, histatin-5, or LL-37 and 0.3 mg/ml TGase were mixed together simultaneously and incubated at 22 °C for various time intervals. The reaction was stopped by addition of 1 mM cysteamine. Samples were subjected to 12% SDS-PAGE, visualized by Coomassie blue or fluorescence and evaluated by densitometry.

### Statistics

Unless specified, all presented data are mean and standard deviation of three independent experiments performed in triplicate. All presented SDS-gels are representative of three independent experiments. Student’s t-test used for calculation of *P*-value.

## Results

### Polymerization of CaATP-G-actin by histatin-3 and histatin-5

**Fig. 1:**
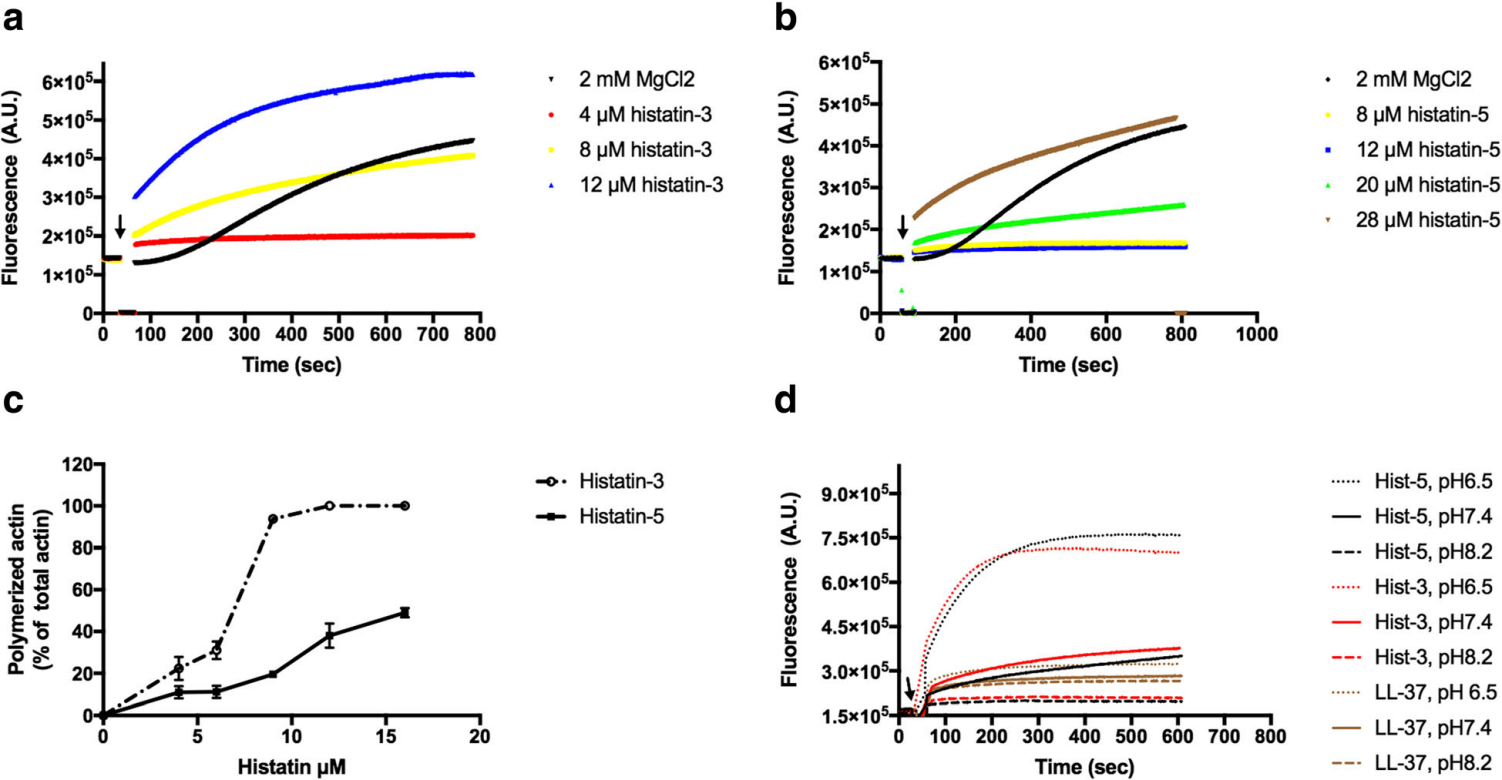
Polymerization of 4 μM CaATP-G-actin by histatin-3 and histatin-5. Pyrene-labeled G-actin was polymerized as described in Methods. Kinetics of polymerization was followed by increase in actin fluorescence. *Arrow* indicates the time of histatin or MgCl_2_ addition **a** Polymerization by histatin-3. **b** Polymerization by histatin-5. **c** Effect of histatin concentration on the extent of polymerization evaluated by densitometry of the SDS-PAGE of supernatants following high speed centrifugation. **d** Kinetics of actin polymerization by 8 μM histatin-3, 20 μM histatin-5 and 7 μM LL-37 at pH 6.5, 7.4 and 8.2

Divalent and polyvalent cations polymerize G-actin to F-actin. The efficiency of polycations as polymerizing agents increases with the number of their net positive charges. We studied the kinetics and extent of actin polymerization by histatin-3 and histatin-5 (Fig. 1). The speed and extent of polymerization was histatin-concentration dependent. However, a considerable difference was found in the ability of the two histatins to polymerize G-actin (Fig. 1a, b and c). Histatin-3 fully polymerized 4 μM G-actin at 12 μM concentration (Fig. 1a and c) but more than 28 μM histatin-5 was needed to achieve a similar degree of polymerization (Fig. 1b). By high speed centrifugation 96 and 20% of F-actin incubated with 9 μM histatin-3 or histatin-5 was sedimented, respectively (Fig. 1c), showing again that histatin-3 is a better polymerizing agent than histatin-5. This is in spite of the fact that both histatins have the same number (5) of net positive charges and that the sequence of histatin-5 is identical with that of histatin-3 in the 1–24 N-terminal amino acids.

The histatin peptides are rich in histidine; both histatin-3 and histatin-5 contain 7 histidine residues. The pK of histidine is around pH 6.5 depending on the environment of the residue in the protein structure. This means that its charge changes with pH in the neutral pH region. Histidine is positively charged at slightly acidic pH and neutral above pH 7.0. Since actin polymerization is dependent on the number of positive charges of the polymerizing agent, the effect of pH 6.5, 7.4 and 8.2 on the polymerization of G-actin by histatins was measured (Fig. 1d). We found that the ability of both histatin-3 and histatin-5 to polymerize actin increased steeply with the decreasing pH. The pH effect on actin polymerization by histatins was compared to its effect on actin polymerization by LL-37, which does not contain histidine residues and at neutral pH polymerizes G-actin at about the same concentration as histatin-3 [9]. The kinetics of actin polymerization by LL-37 was found to be much less pH-dependent than the polymerization by histatins (Fig. 1d).

### Bundling of actin filaments by histatin-3 and histatin-5

**Fig. 2:**
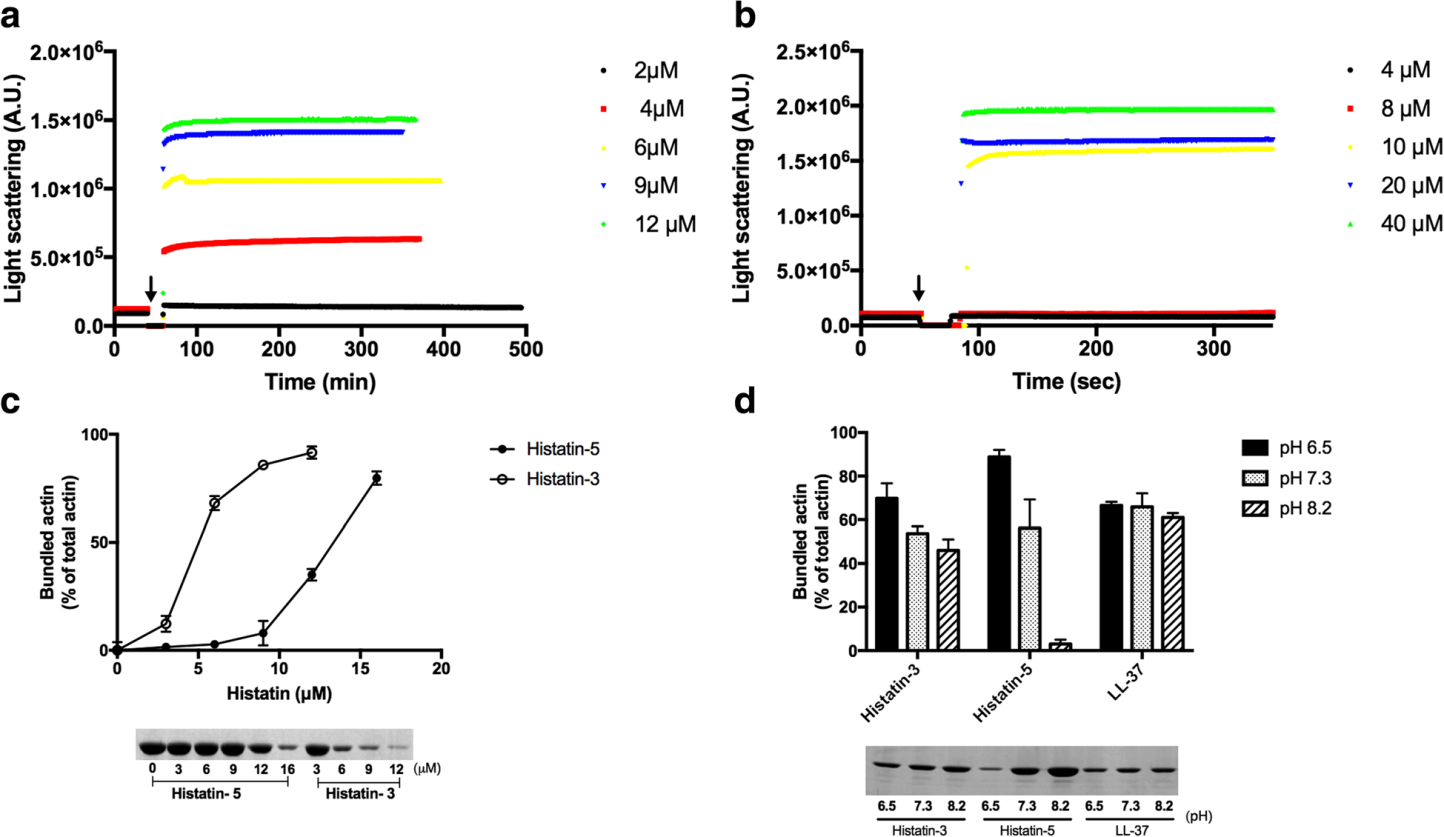
Bundling of F-actin by histatin-3 and histatin-5. Bundling was carried out at low ionic strength. **a** and **b** bundling kinetics of 4 μM F-actin was followed by light scattering change as described in Methods. *Arrow* indicates the time of histatin addition. **a** Bundling by 2–12 μM histatin-3. **b** Bundling by 4–40 μM histatin-5. **c** Extent of bundling of 8 μM F-actin by 0–16 μM histatin-5 and 0–12 μM histatin-3. **d** Extent of bundling of 8 μM Mg F-actin by 5 μM histatin-3, 12 μM histatin-5 and 5 μM LL-37 at pH 6.5, 7.3 and 8.2. **c** and **d** extent of bundling measured by low speed centrifugation and evaluated by densitometry of the SDS-PAGE of supernatants (unbundled actin) as described in Methods. *Upper panels* quantitative evaluation of bundling, *lower panels*, representative SDS-PAGE of the supernatants

Polycations bundle F-actin filaments because their positive charge eliminates the repulsion between the negatively charged actin filaments [13]. We studied the kinetics of F-actin bundling by histatin-3 and −5 by light scattering (Fig. 2a). Histatin-3 and histatin-5 was found to bundle F-actin rapidly at stoichiometric (Fig. 2a), and superstoichiometric concentration (Fig. 2b), respectively. The dependence of the concentration of histatin-3 and −5 on the extent of actin filament bundling was measured by low speed centrifugation (Fig. 2c). We found that histatin-3 is the better bundling agent, since 14 μM of histatin-5, but only 5 μM of histatin-3 were required for 50% bundling 8 μM F-actin.

Bundling of actin filaments, like polymerization, depends on the number of net positive charges in the polycation, which is pH dependent in the histidine rich histatins. The bundling efficiency of histatins, as that of polymerization, was found to increase with the decreasing pH (Fig. 2d). This is contrary to the bundling by LL-37, whose sequence lacks histidine residues. The histatin concentration dependence on the extent of actin filament bundling was measured at pH 6.5 and 7.4 (Additional File 1: Figure S1). At pH 6.5 the bundling takes place at much lower concentrations of histatins than at pH 7.4. The effect of pH on the bundling by histatin-5 is much stronger than by histatin-3 (Fig. 2d and Additional File 1: Figure S1) in spite that both peptides contain the same number of histidine residues and net positive charges.

### NaCl, DNase1 and cofilin unbundle (dissociate) histatin bundled F-actin

**Fig. 3:**
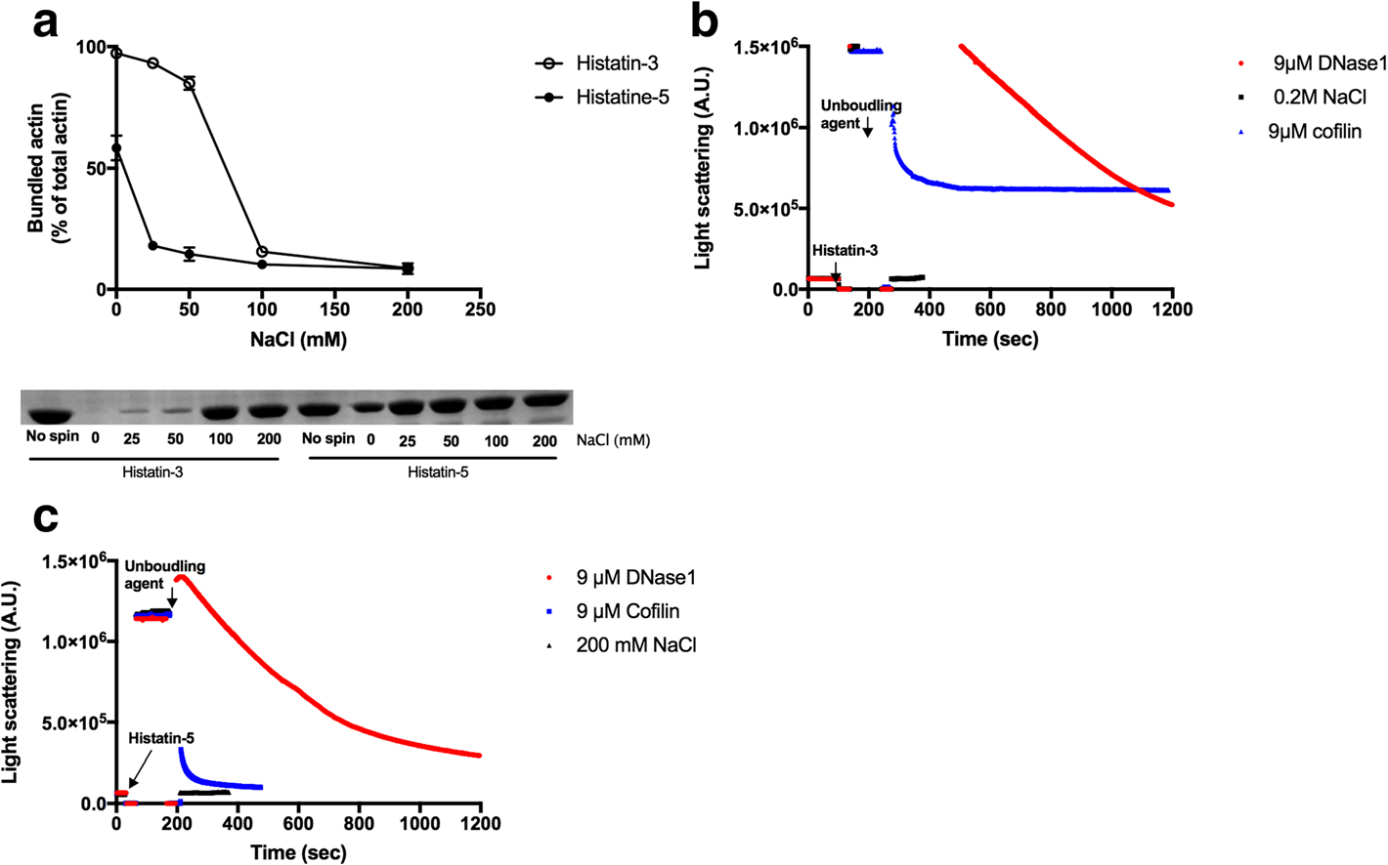
Unbundling (dissociation) of histatin-3 or −5 bundled actin filaments by NaCl, DNase1 and cofilin. **a** Effect of 0–200 mM NaCl on the extent of bundling of 4 μM F-actin bundled by 16 μM histatin-3 or histatin-5 was studied by low speed centrifugation as described in Methods. *Upper panel*, quantitative evaluation of bundling, *lower panel*, representative SDS-PAGE of the supernatants. **b** and **c** Unbundling kinetics by 9 μM DNase1, 9 μM cofilin and 200 mM NaCl of 4 μM F-actin bundled by **b** 8 μM histatin-3 or **c** 16 μM histatin-5. Unbundling was followed by light scattering change as described in Methods

Polycation induced actin bundles are sensitive to ionic strength. They unbundle (dissociate) at increased salt concentration [8] because the salt masks the electrostatic interactions between the polycations and the negatively charged actin filaments [13]. The salt sensitivity of actin bundles is a measure of the relative role of electrostatic and hydrophobic interactions in the binding of polycation to actin. Low ionic strength sensitivity indicates hydrophobic binding between the cationic peptides and actin. Using low speed centrifugation we found that histatin-bundled actin filaments are easily unbundled at relatively low NaCl concentration (Fig. 3a). Bundles induced by 16 μM histatin-5 and histatin-3 are largely dissociated at 25 and 100 mM NaCl concentration, respectively (Fig. 3a). These results indicate that the actin-histatin interactions are basically electrostatic, but, hydrophobic interactions also have minor role in the binding of histatin-3 to actin. The kinetics of bundle dissociation was followed by light scattering, which showed that 200 mM NaCl completely dissociates actin filaments bundled by 8 μM histatin-3 (Fig. 3b) or 16 μM histatin-5 (Fig. 3c) in a few seconds.

DNase1 and cofilin disassemble LL-37 and lysozyme -induced actin bundles [9]. Both DNase1 and cofilin are actin binding proteins. DNase1 depolymerizes F-actin filament by binding to the protomer at the filaments ends and forming a tight complex with the protomer by attachment to its DNase1 binding loop (D-loop). The actin protomer (G-actin)-DNase1 complex gradually dissociates from the end of actin filament as shown by the disassembly of LL-37 induced actin bundles [9]. Cofilin disassembles actin bundles by severing the filaments through its effect on the structure of actin protomers [19, 20]. Light scattering results indicated that 4 μM F-actin bundled by 8 μM histatin-3 (Fig. 3b) or 16 μM histatin-5 (Fig. 3c) dissociates upon addition of 9 μM DNase1 or 9 μM cofilin. The speed of bundle dissociation was fastest with 200 mM NaCl, slower with 9 μM cofilin and slowest with 9 μM DNase (Fig. 3b and c). The light scattering decreased by 60% and 100% after 10 min of addition of 9 μM cofilin to actin filaments bundled by histatin-3 (Fig. 3b) and histatin-5 (Fig. 3c), respectively.

### Effects of histatin-3 and histatin-5 on the fluorescence emission spectrum of TRC and DED labelled F-actin indicate that histatins affect F-actin structure (Fig. 4)

**Fig. 4:**
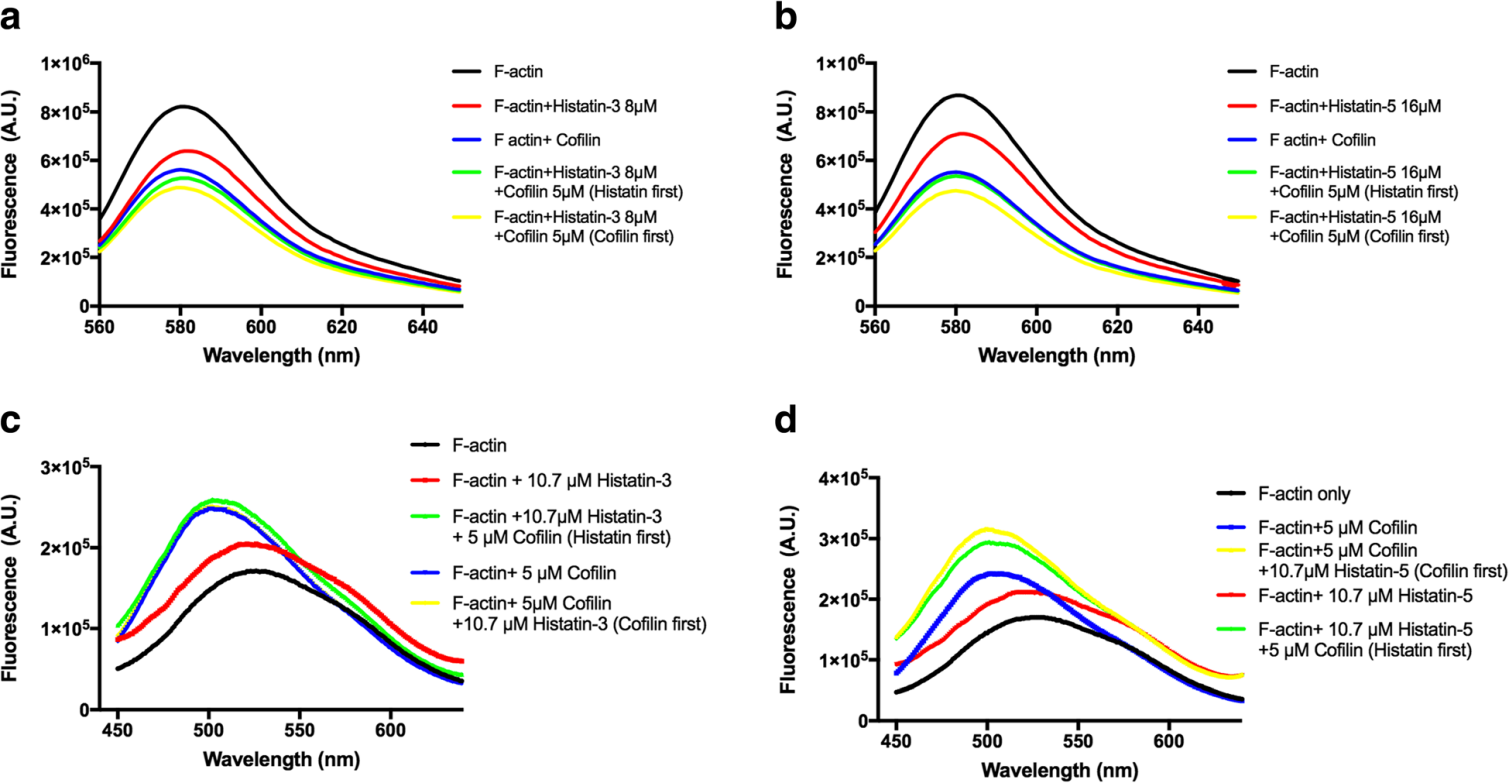
Effect of histatin-3 and histatin-5 on the fluorescence emission spectrum of 5 μM TRC-F-actin and DED-F-actin in the absence and presence of 5 μM cofilin. Fluorescence emission spectra for TRC-F-actin and DED-F-actin were recorded at 544 nm excitation, 560–650 nm emission and 344 nm excitation, 450–640 nm emission wavelengths, respectively. Effect of 8 μM histatin-3 (**a**) and 16 μM histatin-5 (**b**) with and without 5 μM cofilin on the fluorescence emission spectrum of 5 μM TRC-F-actin. Effect of 10.7 μM histatin-3 (**c**), and 10.7 μM histatin-5 (**d**) with and without 5 μM cofilin on the fluorescence emission spectrum of 5 μM DED-F-actin

TRC and DED fluorescence labels bind covalently to Gln-41 in the D-loop of actin in a reaction catalyzed by transglutaminase [20–22]. The fluorescence emission spectra of these labels are sensitive to dynamic changes in actin structure. It was shown that cofilin significantly decreases the fluorescence emission intensity of TRC-labeled [22] and increases the intensity of DED-labeled F-actin [20] due to its effect on the dynamic structure of the D-loop in the subdomain-2 of actin. We found that also histatins affect the fluorescence emission of TRC-F-actin (Fig. 4a and b) and compared these effects with those induced by cofilin. Cofilin-induced fluorescence decrease was found to be significantly greater than that caused by histatins. The effect of histatins and cofilin on the spectrum is additive. Histatin-3 has a larger effect on TRC-F-actin fluorescence than histatin-5, as 8 μM histatin-3 decreases the fluorescence emission at 581 nm (fluorescence emission maximum) by 22.3%, while 16 μM histatin-5 by 18.1%. The effect of histatins and cofilin on the dynamic structure of F-actin was also studied by recording the changes in the fluorescence emission spectrum of DED-label attached to Gln-41 of actin [21]. We found that both histatins and cofilin increase the intensity and blue shift the fluorescence emission spectrum of DED-F-actin (Fig. 4c and d). Both the intensity increase and the blue shift caused by cofilin are significantly greater than those induced by histatins. Histatins move the peak of the spectrum from 528 to 519 nm, while cofilin moves it to 501 nm. The effect of cofilin and histatins on the DED-F-actin spectrum is additive as addition of histatins to cofilin treated F-actin cause further fluorescence intensity increase (Fig. 4c and d). However, the addition of histatin-5 induces much greater increase in the intensity cofilin treated DED-F-actin than that of histatin-3. The histatins and cofilin induced changes in the fluorescence emission spectra of TRC- and DED-F-actin point to alterations in the D-loop structure of actin and in the dynamic structure of F-actin in general. However, there are also differences in histatins and cofilin caused spectral changes, since cofilin induces much larger change in fluorescence intensity in both TRC- and DED-F-actin and significantly greater blue shift in the spectrum of DED-F-actin than histatins do. This indicates that cofilin induces more significant alterations in the dynamic structure of F-actin than histatins. The results also show that the histatin-3 and −5 induced structural changes are not identical as histatin-3 cause larger change in the spectrum of TRC-F-actin than histatin-5, while histatin-5 increases more the emission fluorescence of DED-F-actin in the presence of cofilin than that of histatin-3.

### Histatin-3 and histatin-5 are cross-linked to both G- and F-actin by transglutaminase

**Fig. 5:**
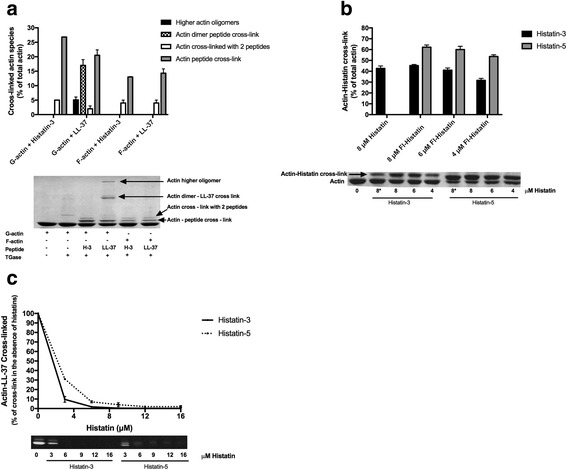
Cross-linking of histatins and LL-37 to CaATP-G-actin and MgF-actin by TGase. Samples were treated with TGase at 22 °C for 30 and run on SDS-PAGE and evaluated by densitometry as described in Methods. *Lower panels* are representative SDS-PAGE-s. *Upper panels* are quantitative evaluation of SDS-PAGE-s. **a** Cross-linking 4 μM G- or F-actin with 8 μM histatin-3 or 6 μM LL-37 by 0.4 mg/ml TGase. **b** Cross linking 4 μM CaATP-G-actin with 8 μM histatin-3 and histatin-5, 4–8 μM Fl-histatin-3, and Fl-histatin-5 by 0.3 mg/ml TGase. Star (*) on 8 in the *lower panel* marks unlabeled histatin. 8 μM histatin-5-actin cross-linked product could not be evaluated accurately because of its poor separation from the actin band. **c** Competition between 6 μM Fl-LL-37 and 0–16 μM histatin-3 or histatin-5 for cross-linking 4 μM CaATP-G-actin. *Lower panel*, SDS-PAGE visualized by fluorescence

Transglutaminase (TGase) catalyzes the formation of isopeptide bonds, which cross-links proximal glutamine and lysine residues in proteins. The TGase reaction produces both intramolecular cross-links in proteins and intermolecular cross-links between proteins and peptides [23]. Transglutaminase treatment of actin has been used to study the structure and function of both monomeric (G) and polymeric (F) actin [21, 24]. We found earlier that LL-37 can be cross-linked to actin by TGase [6, 25]. Here we compared the cross-link formation of histatin-3 and LL-37 with G- and F-actin (Fig. 5a). Histatin-3 was found to be cross-linked to both G- and F-actin like LL-37, but in all cases the extent of cross-link formation with F-actin was significantly less than with G-actin. The bands of a single histatin-3 molecule cross-linked to actin (cross-linked product molecular weight 45 K) and two molecules of histatin-3 cross-linked to actin (48 K molecular weight) were visualized on SDS-PAGE. These products are similar to those obtained by LL-37. However, the bands of actin dimer-peptide and actin higher oligomers-peptide cross-link were missing or weak with histatin-3. We studied the cross-link formation of G-actin also with histatin-5. In this case the actin-single peptide cross-linked band is not well separated from the band of actin because of the low molecular weight of histatin-5 (Fig. 5b). Therefore we used the fluorescence (Fl) derivatives of the histatins for the quantitative study of the cross-linking. With the fluorescence derivatives, the histatin-actin cross-linked bands could be seen without the actin band in fluorescent SDS-PAGE. In Coomassie blue stained SDS-PAGE the separation of Fl-histatin-5-actin monomer cross-linked band from the actin band is better than that of the non-fluorescent histatin-5 because the added mass of the fluorescence moiety (Fig. 5b). The extent of cross-linking is slightly larger with the fluorescent than with the non-fluorescent histatins. Comparing the cross-linking of Fl-histatin-3 and Fl-histatin-5 with G-actin we noticed that the quantity of the Fl-histatin-5-actin cross-linked product is consistently greater than that of Fl-histatin-3 (Fig. 5b). To compare histatin-actin cross-links with LL-37-actin cross-link histatin-3 and −5 were competed with Fl-LL-37 for cross-linking to G-actin (Fig. 5c). We found that 3 μM histatin-3 or histatin-5 decreased the extent of cross-linking of 4 μM G-actin with 6 μM Fl-LL-37 by 92 and 68% percent, respectively. The findings that at substoichiometric concentrations of histatins relative to Fl-LL-37, inhibited more than 50% of the cross-linking of Fl-LL-37, indicate that the ability of histatins to cross-link G-actin is greater than that of LL-37. Since LL-37 binds preferentially to the D-loop of actin [6], the competition of histatins with LL-37 for actin cross-linking may suggest that histatin-3 and −5 also bind to the same actin loop.

### Kinetics of cross-link formation and the effect of phalloidin on the extent of cross-link between actin and Fl-histatins

**Fig. 6:**
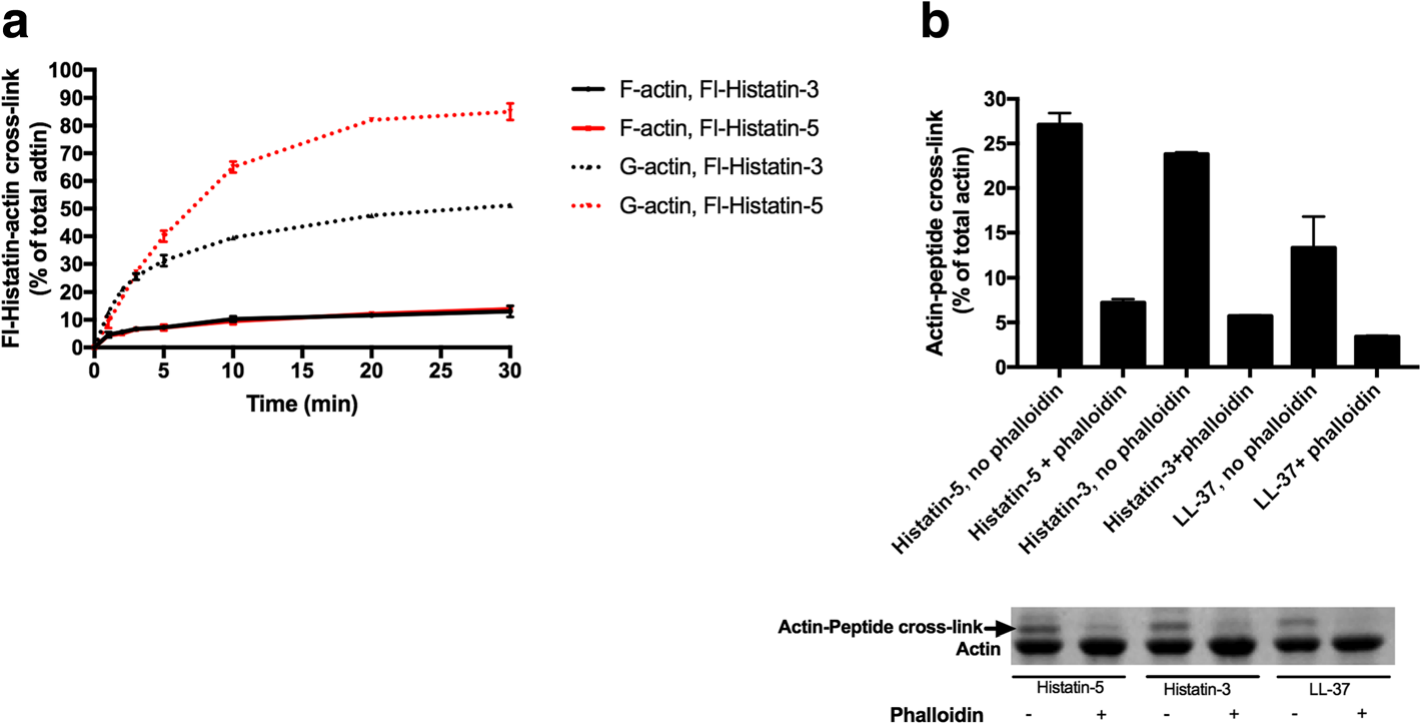
Kinetics of cross-linking of Fl-histatin-3 and Fl-histatin-5 to G- and F-actin and effect of phalloidin on the extent of cross-linking of Fl-histatin-3, Fl-histatin-5 and Fl-LL-37 to F-actin. **a** Kinetics of cross-linking of 6 μM Fl-histatin-3 and −5 to 4 μM F-and G-actin. Samples were treated with 0.3 mg/ml TGase at 22 °C for various time intervals, run on SDS-PAGE and evaluated by densitometry. **b** Effect of 8 μM phalloidin on the extent of cross-linking of 8 μM Fl-histatin-3, Fl-histatin-5 and Fl-LL-37 to 4 μM F-actin. Cross-linking with 0.3 mg/ml TGase for 30 min at 22 °C. *Upper panel*, quantitative evaluation of SDS-PAGE by densitometry. *Lower panel* Coomassie blue stained representative SDS-PAGE

The kinetics (Fig. 6a) and extent (Fig. 6b) of the TGase catalyzed cross-link formation of Fl-histatins to G- and F-actin was studied. The extent of cross-linking of Fl-histatin-5 to both G- and F-actin was larger than that of Fl-histatin-3 (Figs. 5b and 6). The cross-linking of Fl-histatins to G-actin was faster and its extent significantly larger than to F-actin (Fig. 6). As the result of treadmiling [26] there is always a small concentration of G-actin in F-actin preparation and equilibrium exist between the two forms. Because of the large difference in the extent of cross-linking of histatins to G- and F-actin one may assume that also in the case of F-actin, the G-actin, which is present in the F-actin preparation, reacts with histatins. The actual cross-linking of histatins to F-actin might be rather minor. To check this assumption we cross-linked F-actin with histatins and LL-37 also in the presence of phalloidin (Fig. 6b). Phalloidin inhibits treadmiling and decreases the concentration of G-actin present in the F-actin preparations [27]. We found that phalloidin reduces greatly the extent of F-actin cross-linking with Fl-histatin-3, Fl-histatin-5 and Fl-LL-37 (Fig. 6b), which supports the above hypothesis. The poor cross-linking of histatins and LL-37 to F-actin is caused by the structural changes taking place in the D-loop of actin during polymerization.

### NaCl, DNase1 and cofilin inhibit the cross-linking of histatins to G-actin

**Fig. 7:**
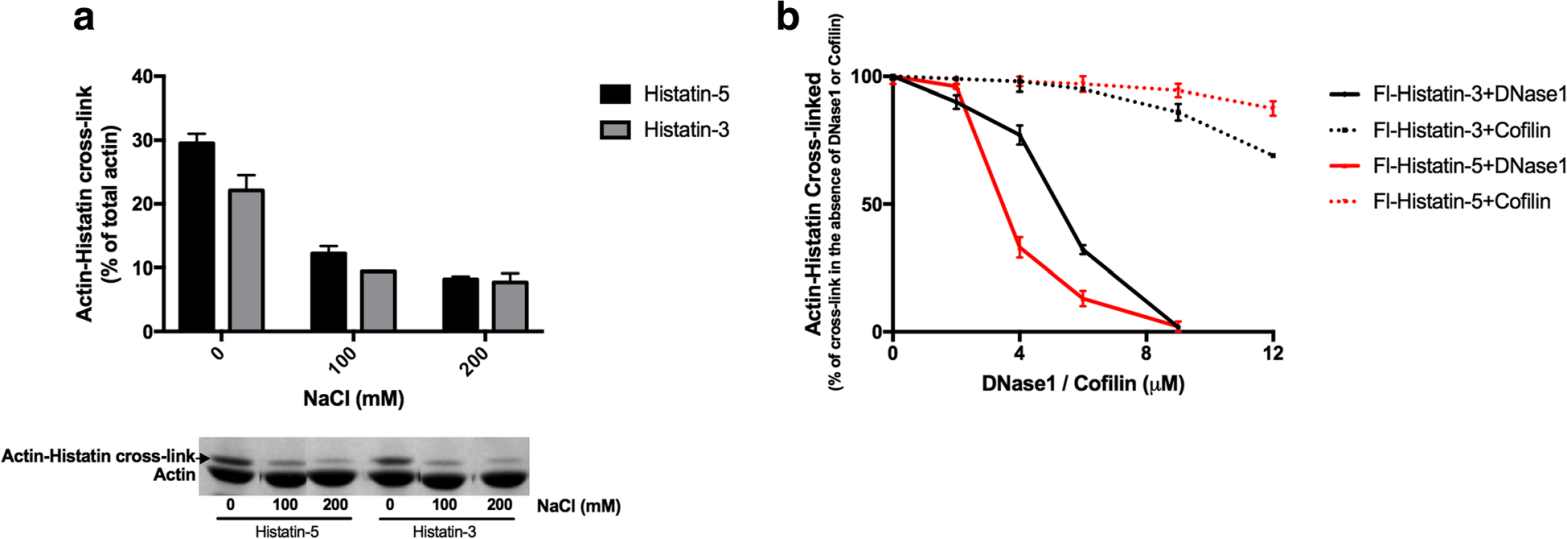
Inhibition of cross-linking of 6 μM Fl-histatin-3 and Fl-histatin-5 to 4 μM G-actin by NaCl, DNase1 and cofilin. Samples were treated with 0.3 mg/ml TGase at 22 °C, run on SDS-PAGE and evaluated by densitometry. **a** Inhibition of cross-linking by NaCl. Samples were treated with TGase for 1 min. *Upper panel*, evaluation of SDS-PAGE. *Lower panel*, representative SDS-PAGE visualized by Coomassie blue. **b** Inhibition of cross-linking by DNase1 and cofilin. Samples were treated with TGase for 5 min

We found that NaCl, DNase1 and cofilin unbundles histatin-3, histatin-5 (Fig. 3) and LL-37 [9] induced F-actin bundles. Here we studied the effect of these agents on the cross-link formation between G-actin and histatins. In these experiments NaCl, histatins, TGase were added simultaneously to G-actin and incubated for 1 min only to avoid significant actin polymerization. NaCl inhibited the cross-linking of Fl-histatin-3 and −5 to G-actin (Fig. 7a), but its effect was less than that on dissociation of bundles (Fig. 3). Addition of 200 mM NaCl decreased the extent of cross-linking by histatins more than 70% (Fig. 7a) but did not affect the extent of cross-linking by LL-37 [6]. These results support the conclusions of the unbundling experiments with NaCl (Fig. 3a) that the binding of histatins to actin is less hydrophobic than that of LL-37.

DNase1, which binds to D-loop of actin, strongly inhibits cross-link formation between G-actin and histatins (Fig. 7b). The strong inhibitory effect of DNase1 on the histatin-actin cross-linking indicates (see also Fig. 5b) that histatin 3 and −5 bind to the DNase1 binding loop (D-loop) of G-actin. Cofilin, which partially binds to the DNase binding loop actin [28], only slightly inhibits the Fl-histatin-G-actin cross-linking (Fig. 7b). The inhibitory effect of cofilin was less Fl-histatin-5 than with Fl-histatin-3.

### Histatin cross-linking sites on G-actin

**Fig. 8:**
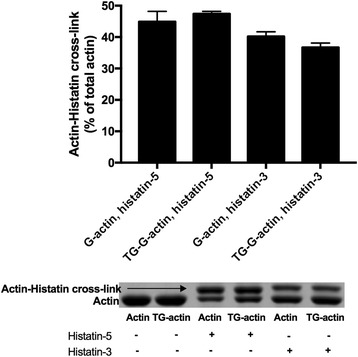
Cross-linking of 4 μM CaATP-G-actin and TGase pretreated CaATP-G-actin (TG-G-actin) with 4.5 μM Fl-histatin-3 and Fl-histatin-5 by 0.3 mg/ml TGase at 22 °C for 30 min. Preparation of TG-G-actin: 108 μM CaATP-G-actin was incubated with 4 mg/ml TGase at 4 °C for 24 h. After cross-linking samples were run on SDS-PAGE and evaluated by densitometry. *Upper panel* quantitative evaluation of SDS-PAGE by densitometry. *Lower panel* representative SDS-PAGE visualized by Coomassie blue

TGase catalyzes covalent cross-linking of glutamine and lysine residues in proteins [23]. Since in the histatin-G-actin cross-linking reaction only actin contains glutamine residues, it follows that actin should be the glutamine donor in this case. DNase1, which binds to the D loop (His40-Lys50 sequence) of G-actin [29], strongly inhibits cross-linking of histatins to actin (Fig. 7b). These results indicate that histatin-3 and −5 also bind to the D-loop of G-actin and should be cross-linked to either Gln41 or Gln49 in the D-loop. Gln41 seems to be the preferred candidate for this role since this residue participates in the intrastrand cross-linking by N-(4-azido-2-nitrophenyl) putrescine between F-actin protomers [30] and in the intramolecular actin cross-linking to Lys50 [24]. This residue was also labeled with DED [21] and TRC [22] fluorescent probes by TGase. On the other hand LL-37, which also binds to the D-loop [6], is preferentially cross-linked to Gln-49 [6]. The cross-linking of Fl-LL-37 to G-actin is inhibited by histatin-3 and −5 (Fig. 5c). To locate the cross-linking site in the D-loop we cross-linked G-actin and TGase pretreated G-actin (TG-G-actin) with Fl-histatin-3 and −5 (Fig. 8). The TGase treatment of G-actin yields intramolecular cross-link between Gln41 and Lys50 and makes Gln41 unavailable for further cross-linking [24]. We found that slightly less actin Fl-histatin-3 cross-linked products formed with TGase pretreated than with untreated G-actin. (Fig. 8). Therefore, the result that TGase pretreatment only slightly decreases the cross-link formation between G-actin and Fl-histatin-3 indicates that Gln-49 is the main glutamine donor in the D-loop of actin in this reaction. However, a minor role of Gln41 cannot be excluded in this cross-linking. On the other hand, the same amount of cross-linked product formed in the reaction of G-actin or TG-G-actin with Fl-histatin-5 (Fig. 8) indicating that Fl-histatin-5 does not cross-link to Gln41 of G-actin.

**Fig. 9:**
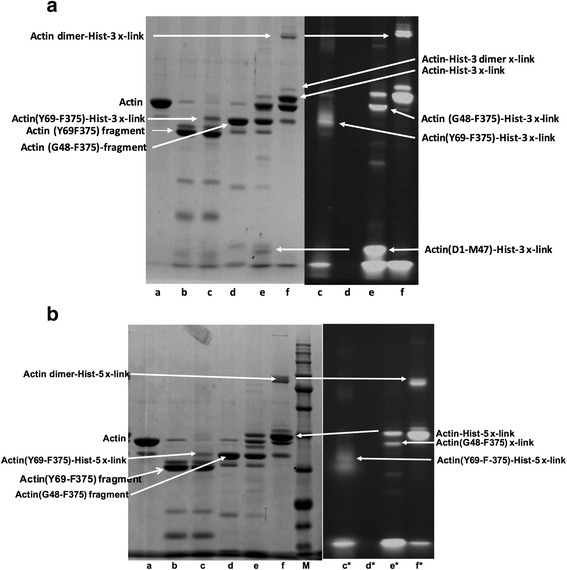
4 μM CaATP-G-actin was digested by 1.5 μg/ml subtilisin or 20 μg/ml trypsin at 22 °C for 10 min and was cross-linked to 6 μM Fl-histatin-3 and Fl-histatin-5 by 0.3 mg/ml TGase at 22 °C for 30 min. Trypsin and subtilisin digestions were stopped by 40 μg/ml STI and 1 mM PMSF, respectively. Samples were run on SDS-PAGE. *Left*, lanes visualized by Coomassie blue; *right*, lanes visualized by fluorescence, marked by (*). Legend: (a) actin; (b) trypsin digested actin; (c) trypsin digested actin cross-linked with Fl-histatin-3 or Fl-histatin-5; (d) subtilisin digested actin; (e) subtilisin digested actin cross-linked with Fl-histatin-3 or Fl-histatin-5; nf), actin cross-linked with Fl-histatin-3 or Fl-histatin-5. **a** Fl-histatin-3-actin fragments cross-linked bands; **b** Fl-histatin-5-actin fragments cross-linked bands. Lane M, molecular weight marker

Subtilisin cleaves G-actin into Asp1-Met47 N-terminal and Gly48-Phe375 C-terminal fragments [31]. We found that both fragments can be cross-linked with Fl-histatin-3 (Fig. 9a). The N-terminal fragment contains Gln41 as single glutamine residue which proves that Gln-41 can also be glutamine donor in cross-linking of Fl-histatin-3 to G-actin. However, Fl-histatin-5 is cross-linked only to the C- but not to the N-terminal G-actin fragment (Fig. 9b), which supports our finding that Fl-histatin-5 is not cross-linked to Gln41 of G-actin (Fig. 8). The cross-link formation between Fl-histatin-3 or Fl-histatin-5 and the C-terminal actin fragment (Fig. 9), which contains the other D-loop glutamine, Gln49, indicates that this residue is the main cross-linking site on the D-loop of G-actin. Trypsin cleaves G-actin between Lys68 and Tyr69 leading to the formation of stable Tyr69-Arg372 C-terminal fragment [31]. We found that the Tyr69-Arg372 C-terminal tryptic fragment, which does not contain the D-loop, can be also cross-linked to both Fl-histatin-3 and −5 (Fig. 9). This finding indicates that beside Gln41 and Gln49 other, non-D-loop glutamines of actin, can also serve as minor glutamine donors in the TGase catalyzed actin-histatin cross-linking reaction.

## Discussion

Histatin-3 and histatin-5 are histidine-rich polycationic peptides; they polymerize G-actin to F-actin filaments and induce rapid bundling of the filaments. The polymerization and bundling of actin by histatins is pH dependent. The binding of histatins to actin increases with decreasing pH. This can have biological relevance since the pH of saliva is changes between pH 6.2 and 7.5 [34]. NaCl dissociates the histatin induced actin bundles similar to observed also with the polylysine, lysozyme [8] and LL-37 [9] polycations. NaCl increases ionic strength and dissociates bundles by masking the electrostatic interactions between the polycationic histatin and the negatively charged actin filaments [13]. The dissociation of the histatin induced actin bundles at relatively low ionic strength indicates that histatins are bound to F-actin by mainly electrostatic interactions. Histatin-induced bundles are also unbundled by DNase1, which dissociates actin monomers from the end of the filaments [32]. Finally, cofilin dissociates histatin-F-actin bundles by severing F-actin to shorter filaments [33].

Higher concentration of histatin-5 is needed to polymerize G-actin and to bundle F-actin than that of histatin-3. The sensitivity of histatin-5 bundled F-actin to ionic strength is much greater than that of histatin-3 bundled F-actin. The dissociation of histatin-5 bundled actin filaments is also faster and more complete with DNase1 and cofilin than those bundled by histatin-3. The polymerization and bundling of actin by histatins are pH sensitive because of their high histidine content. The pK of histidine residues is around pH 6.5 and their positive charge increases with the decreasing pH. The extent of bundling of F-actin by histatin-5 increases more with the decreasing pH than by histatin-3 (Fig. 2d). The effect of pH on the bundling of histatin-3 and −5 is different in spite of their almost identical amino acid sequence and equal number of net positive charges (5) and histidine residues (7). The only difference between histatin-3 and −5 is that the C-terminal RSNYLYDN segment (which contains one positive (R) and one negative (D) charges, but no net charge) of histatin-3 is missing from the sequence of histatin-5. It seems that this C-terminal segment of histatin-3 stabilizes the F-actin bundles against dissociation by increasing ionic strength, DNase1 and cofilin and decreases its pH sensitivity. The possible reason of the different pH sensitivity is that the extra C-terminal segment in histatin-3 shifts the pK of the histidine residues to higher pH. Therefore, at neutral pH histatin-3 is more positive charged than histatin-5 and its binding to the anionic F-actin is stronger than that of histatin-5. Hydrophobic interactions may exist between the extra C-terminal sequence of histatin-3 and F-actin, which could also contribute to the relatively strong binding of the histatin-3 to actin. The results indicate that the effect of polycationic peptides on actin polymerization and bundling depends both on their net positive charges and amino acid sequence.

Histatin-3 and −5 affect the fluorescence emission spectra of the TRC and DED fluorescence probes, which are covalently bound to Gln41 in the D-loop of F-actin. The spectral changes indicate that histatins affect the dynamic structure of the D-loop in F-actin. The spectral alterations were different with histatin-3 as with histatin-5 indicating that the effect of the two histatins on actin structure is not identical. The histatins induced spectral changes are similar but smaller than those obtained upon cofilin addition to F-actin [20, 22] suggesting that the histatins induced dynamic structural changes in the D-loop of F-actin resemble to those caused by cofilin.

Histatin-3 and histatin-5 are cross-linked to both G- and F-actin by transglutaminase. The cross-linking to G-actin takes place faster and with a higher yield than to F-actin. The cross-linking of histatins to F-actin might be an artifact and takes place because the small concentration of G-actin present in in the F-actin preparation. The cross-linking of histatin-5 to G-actin is faster and resulting higher yield than that of histatin-3. The cross-linking of histatins to G-actin is inhibited by DNase1 (Fig. 9b), which binds to the D-loop of G-actin. Histatins compete for cross-linking to G-actin with LL-37 (Fig. 7b), which is cross-linked to Gln-49 in the D-loop of G-actin [6]. These findings suggest that the histatins bind to the D-loop of G-actin. This assumption is supported with the results of limited proteolysis of histatin cross-linked G-actin with subtilisin and trypsin (Fig. 9), which showed that the main cross-linking site in the D-loop of G-actin is Gln-49.

## Conclusions

Both histatin-3 and −5 interact with actin. However, there are significant differences in their interactions with actin in spite of the identical number of positive charges and histidine residues and nearly identical sequence of the two histatins. The interaction of histatin-3 and histatin-5 with actin is mainly electrostatic but that of histatin-3 is partially hydrophobic. A higher concentration of histatin-5 than histatin-3 is needed for the polymerization and bundling of actin and their sensitivity to factors affecting interactions with actin is also different. These differences derived from the extra 8 amino acid sequence at the C-terminus of histatin-3, which is missing from histatin-5, and also reflected in the biological activity of the two. While both histatin-3 and −5 demonstrated candidacidal activities, they differed in their abilities to kill blastoconidia and germinated cells, with histatin-5 being the more active than histatin-3. For the inhibition of germination, however, histatin-3 exhibited more activity than histatin-5 [2]. The interactions of histatins with actin have biological relevance because actin, as histatins, is found in the saliva [6]. The pH of saliva is in the pH 6.2–7.5 range [34]. The polymerization and bundling of actin by histatins is pH dependent and increases with decreasing pH. Thus actin-histatin interactions are more relevant in slightly acidic pH saliva. Actin is released into the saliva from necrotic cells during oral inflammatory infection. It is therefore possible that the released actin might have an effect (most likely deleterious) on the health-promoting activity of salivary histatins. This should be the subject of further studies.

## Additional file

Additional File 1: Figure S1. Effect of pH on bundling. 8 μM F-actin was bundled by 3–12 μM histatin-3 or 3–16 μM histatin-5 at pH 6.5 and 7.4. Extent of bundling measured by low speed (20,800xg for 8 min) centrifugation and evaluated by densitometry of the SDS-PAGE of supernatants (unbundled actin) as described in Materials and Methods. (TIFF 192 kb)

## Abbreviations

ATCUN tripeptide: Amino Terminal Copper and Nickel
DED: Dansyl ethylenediamine
DNase1: Deoxyribonuclease 1
DTT: Dithiotreitol
eEF1A: Eukaryotic elongation factor 1 alpha
F-actin: Filamentous actin
Fl: Fluorescence
Fl-histatin-3: Tetramethylrhodamine labeled histatin-3
Fl-histatin-5: FAM labeled histatin-5
Fl-LL-37: Tetramethylrhodamine labeled LL-37
G-actin: Globular actin
MOPS: 3-(N-morpholino) propane sulfonic acid
SDS-PAGE: Sodium dodecyl sulfate polyacrylamide electrophoresis
TGase: Bacterial transglutaminase
TRC: Tetramethyl rhodamine cadaverine
