# Supplementary figures and images for: Interactions of histatin-3 and histatin-5 with actin
Source: BMC Biochem. 2017 Mar 6;18:3. doi: 10.1186/s12858-017-0078-0 (PMC5340040; doi:10.1186/s12858-017-0078-0)

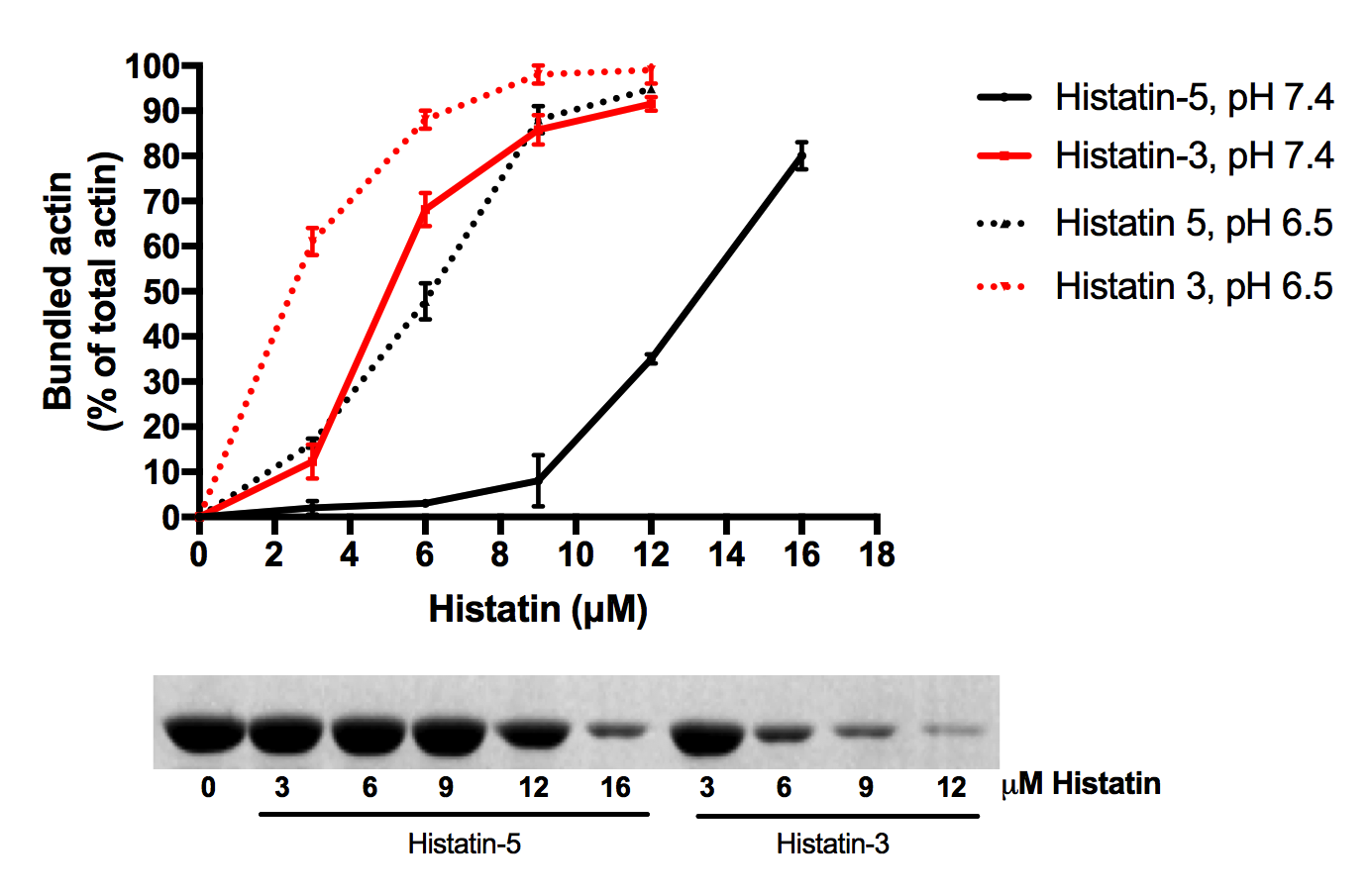

Supplement: Additional File 1: Figure S1. — Effect of pH on bundling. 8 μM F-actin was bundled by 3–12 μM histatin-3 or 3–16 μM histatin-5 at pH 6.5 and 7.4. Extent of bundling measured by low speed (20,800xg for 8 min) centrifugation and evaluated by densitometry of the SDS-PAGE of supernatants (unbundled actin) as described in Materials and Methods. (TIFF 192 kb) [file 12858_2017_78_MOESM1_ESM.tiff]
